# The use of internal irrigation versus external and combined irrigation during dental implant bed preparation, a systematic review

**DOI:** 10.1186/s40729-026-00688-0

**Published:** 2026-05-08

**Authors:** Valerio Cimini, Alexandre Perez, Hamzah Shabana, Roberto Di Felice, Tommaso Lombardi

**Affiliations:** 1 Unit of Oral Surgery and Implantology, Division of Oral and Maxillofacial Surgery, Department of Surgery, Faculty of Medicine, University Hospitals of Geneva, University of Geneva, 1205 Geneva, Switzerland; 2 Dental Clinic and Research Center San Benedetto del Tronto, Tronto, Italy; 3 Unit of Oral Medicine and Oral Maxillofacial Pathology, Division of Oral and Maxillofacial Surgery, Department of Surgery, Faculty of Medicine, University Hospitals of Geneva, University of Geneva, 1205 Geneva, Switzerland

**Keywords:** Dental implants, Implant bed preparation, Implant placement, Cooling, Bone heating, Internal irrigation, External irrigation

## Abstract

**Purpose:**

The aim of this study was to analyze the role and capacity of internal irrigation compared to external irrigation and, when applicable, combined irrigation to reduce and control bone heating during implant bed preparation.

**Methods:**

This systematic review was conducted according to predefined inclusion and exclusion criteria. A comprehensive literature search was performed in PubMed, Embase, Cochrane, Web of Science, and Google Scholar up to September 2025. Studies evaluating irrigation methods during implant osteotomy were included. Screening, selection, and risk of bias assessment were performed following a standardized protocol.

**Results:**

201 records were found. After deduplication, screening, and full-text evaluation, 10 in vitro studies were included for qualitative analysis and evaluation of risk of bias. At the same time, no animal studies fully met the inclusion criteria, and no randomized clinical trials were identified. Reports show that the peak temperature changes (ΔT) varied substantially, from 1.48 to 75.4 °C.

**Conclusion:**

In most studies, internal irrigation was shown to have superior cooling efficiency during the implant bed preparation compared to external irrigation, particularly in deeper osteotomies or when surgical guides were used. Combined irrigation also appeared to be a beneficial method. However, its performance varied depending on drill design and experimental setup. Risk of bias, assessed with the QUIN tool, was moderate to high, reflecting heterogeneity in study model, sampling, and reporting. Within the limitations of current evidence, internal irrigation emerges as an efficient method to control thermal and heat elevation during implant osteotomy.

## Introduction

Dental implants have proven to be a highly successful therapeutic option for single tooth replacement, with reported success rates ranging from 96.7 to 100% within an average follow-up period of 29 months [1] for the replacement of single missing teeth.

The successful integration of the implant and bone without the interposition of soft tissue, called osseointegration, is based on the principle of bone regeneration [2].

Failure of osseointegration leads to what is referred to in the literature as "early failure" [3].

Several factors cited in the literature may interrupt osseointegration, such as the quality of the implant site bone, surgical protocols, medications, implant biomechanics, and the patient’s health status [1, 2, 4].

Despite the consensus that in some cases cell death during surgery is inevitable [5], preserving bone vitality is crucial to ensure osseointegration [6] and implant success.

During implant surgery, the implantation site must be prepared before placing the implant, including the creation of the surgical flap and the drilling of the bone [7].

The temperature at the implant site can however rise significantly during drilling due to the combined effects of friction between the drill bit and the bone, drill speed and the length of time spent preparing the site.

Elevated temperatures can severely impact human bone vitality, potentially causing bone necrosis. Surgical protocols have identified heat generation as a critical factor influencing the success of primary implant integration and bone viability [8].

It is well known that the phenomenon of increased temperature at the implant site leads to osteonecrosis. Eriksson et al. [6] conducted two studies on animal bone demonstrating that bone is sensitive to temperatures around 47 °C, and heating at 53 °C for one minute interrupts blood circulation in the bone and results in irreversible bone damage [4, 5]. This can lead to crestal bone loss during the healing phase [7]. However, the exact temperature of thermal necrosis during drilling the implant site is still unknown [9].

Regarding conventional drilling, procedures related to temperature development, drill speed, time of exposure, applied pressure, drilling motion pattern, bone density [6, 10, 11], drilling depth [12], and irrigation [13, 14] have already been described in the literature [15, 16]. The variability in drill design also appears to play a role, including the number of drill blades, drill design (conical or straight) [17], drill fatigue, thermal capacity, and thermal conductivity [9, 10, 12, 18].

One of the methods used to control temperature during drilling is the use of abundant irrigation during implant site preparation [19].

Since Hippocrates [20], who was among the first promoting irrigation to prevent heat-induced bone tissue injuries, it has long been recognized that adequate saline irrigation during osteotomies is essential to avoid the destruction of the osteointegration.

Irrigation methods reported in the literature include conventional external irrigation, where the fluid is sprayed onto the drill from an external injection, and internal irrigation, whereby fluid is delivered with an internal hole in the drill [21]. In general, two types of irrigation are common in implant dentistry during the preparation of the implant site, namely external and internal irrigation. Most published studies have focused on temperature changes during drilling an implant site and osteotomy, with particular focus on external irrigation systems [21].

External irrigation is mainly effective in the superficial cortical bone regions, while internal irrigation is thought to be more advantageous in deeper osteotomies for minimizing friction-induced heat [22]. To the best of our knowledge, internally cooled drills or internal irrigation and reamers were first introduced to implant dentistry in 1975 by Kirschner and Meyer [23]. Since the cooling saline water is released directly from the drill tip, these instruments are supposed to provide better cooling and rinsing during the implantation compared to externally cooled drills.

The use of a combined irrigation system can effectively reduce temperature and heat production during drilling. It appears to be extremely helpful for deeper osteotomies, where precise thermal control is critical and needed for preserving bone integrity [24].

Our definition of internal irrigation is the presence of a channel of irrigation inside the implant preparation drill. Frequently, the term is used to describe also the presence of an additional irrigation channel when using a surgical guide. This difference was strictly observed by the researchers.

The present review aimed to analyze the efficacity of internal irrigation during implant placement compared to external irrigation and, when present, combined irrigation.

During our systematic review, both irrigation methods will be evaluated to create a comparative table aimed at determining which method is more effective or preferable to use in clinical practice.

## Materials and methods

### The identifying question

Is internal irrigation as effective as external or combined irrigation in controlling temperature at the implant bed site during implant placement?

### A search strategy

First, the study was registered on the OSF database, and it could be found at the link: https://osf.io/mcskh/?view_only=517d4d71d7b74a94a51db11bb90b8c4a.

This study was designed following the PRISMA (Preferred Items for Reporting Systematic Reviews and Meta-Analyses) guidelines and the PRISMA statement was checked and sent to editor [25].

The PICO strategy was designed as described: surgical procedure of implant bed preparation in humans, animals and samples (P) using the internal irrigation (I). The main comparison (C) was with the correspondent method used in literature, that is the external irrigation and combined irrigation. The outcomes (O) were the temperature variations and the final temperatures that can be classified as main outcome.

For this reason, the Mesh was designed following the concepts (P) and (I), i.e., the population and intervention. This allowed for a wide choice of articles and opened possibilities for different outcomes.

The inclusion criteria comprised all the in vitro, animal and clinical studies published in English that discussed and performed implant bed preparation using internal irrigation in comparison with one or more groups of different irrigation conditions.

We have selected RCTs, prospective or retrospective studies, case series with at least 5 cases, and observational trials.

The Systematic reviews that were found were included using the snowball method to extract individual articles that met our inclusion criteria. Even during the search, backward and forward snowballing was performed to add all the matching articles.

The exclusion criteria included all articles such as letters to editors, case reports, case series with less than 5 cases, narrative reviews, congress abstracts and opinion articles. Also, articles performing surgical procedures other than implant bed preparation using instruments other than drills were excluded.

The search was performed on Pubmed, Embase, Cochrane and Web of Science databases. The medical subject heading terms used for the research combined the two concepts of implant bed preparation and internal irrigation. The complete MeSH is structured as follows:

**Table:** 

Pubmed	("Dental Implants"(MeSH Terms) OR "Dental Implantation"(MeSH Terms) OR "dental implantation/methods"(MeSH Terms) OR "dental implant*"(Title/Abstract) OR "implant bed prepar*"(Title/Abstract) OR "implant osteotom*"(Title/Abstract) OR "implant placement*"(Title/Abstract) OR "implant prepar*"(Title/Abstract) OR "implant site drill*"(Title/Abstract)) AND ("internal cooling"(Title/Abstract) OR "internal irrigat*"(Title/Abstract)
Embase	('tooth implant'/de OR ((implant* NEAR/2 (bed OR prepar* OR ostetom* OR placement* OR site OR dental OR drill*)):ab,ti,kw)) AND ('cooling water'/de OR 'cooling'/de OR ((intern* NEAR/2 cool*):ab,ti,kw) OR (((drill* OR intern*) NEAR/2 irrigation):ab,ti,kw))
WOS	(implant* NEAR/2 (bed OR prepar* OR ostetom* OR placement* OR site OR dental OR drill*)) AND ("cooling water" OR (intern* NEAR/2 cool*) OR ((drill* OR intern*) NEAR/2 irrigation))
Cochrane	(implant* NEAR/2 bed OR implant* NEAR/2 prepar* OR implant* NEAR/2 ostetom* OR implant* NEAR/2 placement* OR implant* NEAR/2 site* OR implant* NEAR/2 dental OR implant* NEAR/2 drill*) AND ("cooling water" OR intern* NEAR/2 cool* OR drill* NEAR/2 intern* OR drill* NEAR/2 irrigation)

To investigate the grey literature a parallel manual search on Google scholar was performed. The free terms were used as a combination of concepts P and I.

The main objective was to study the efficacy of controlling bone temperature during bone preparation, which is one of the most important aspects for bone healing during this surgical procedure.

The articles were selected without any limitation of time until September 2025, date of extrapolation of data.

After data extraction, deduplication using the “Deduplicator” tool from Systematic Review Accelerator (https://sr-accelerator.com) was performed.

Title and abstract screening were performed by H.S. and V.C., respectively. All articles selected in this way were retrieved for full-text analysis.

**Fig. 1:**
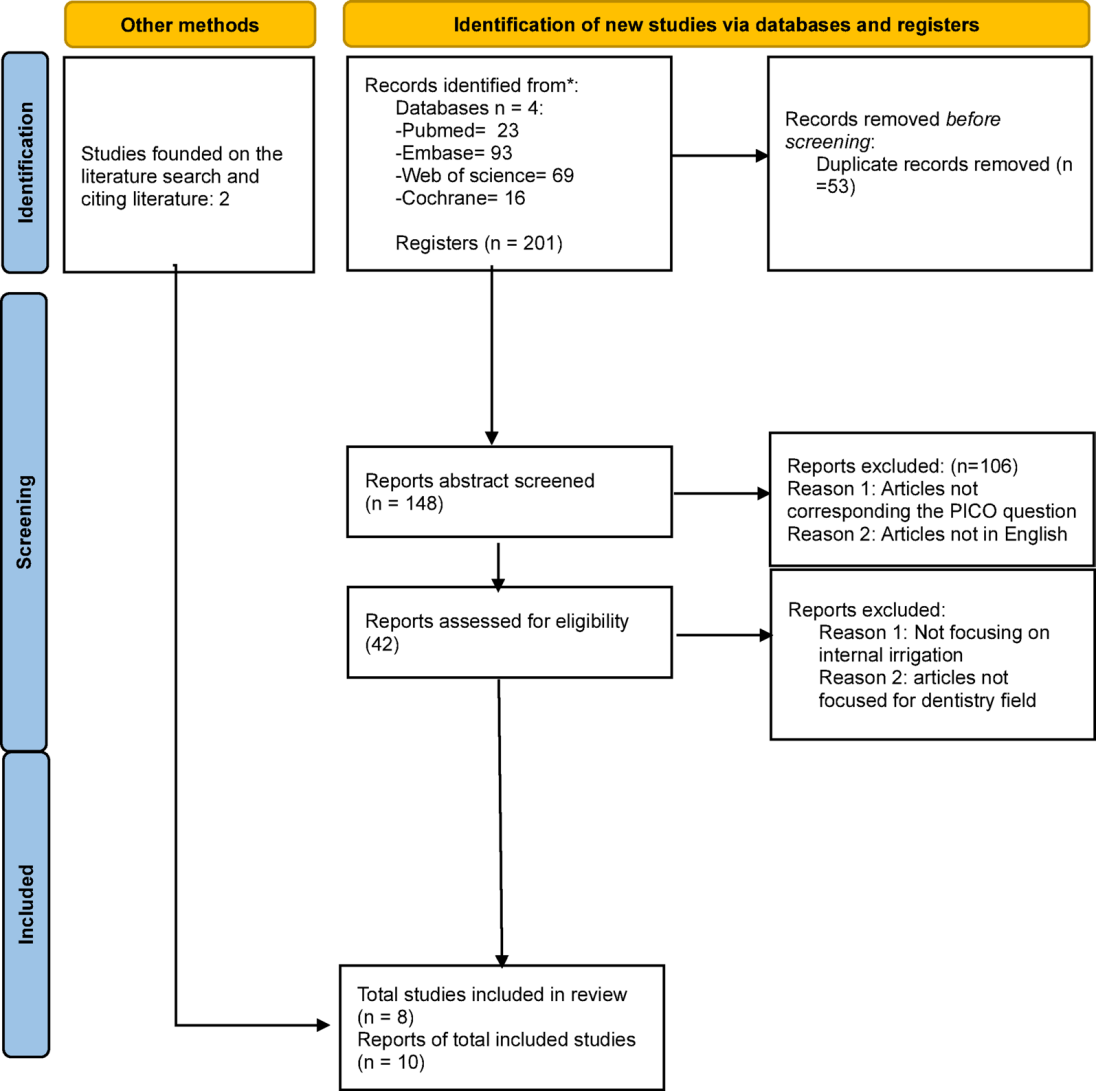
PRISMA flow chart and number of results for each step of the research

In case of disagreement between the reviewers, a third reviewer (T.L.) was consulted to resolve the issue and provide an opinion. The PRISMA flow chart shows the results and the numbers for all stages of the research process (Fig. 1).

### Risk of bias

To assess the risk of bias we used the QUIN checklist [26], and we calculated the risk from low to high using the 12-tool system. Following the instructions, when possible, a score between 0 and 2 was assigned to each item. Items that were not applicable were marked as ‘NA’.

**Fig. 2:**
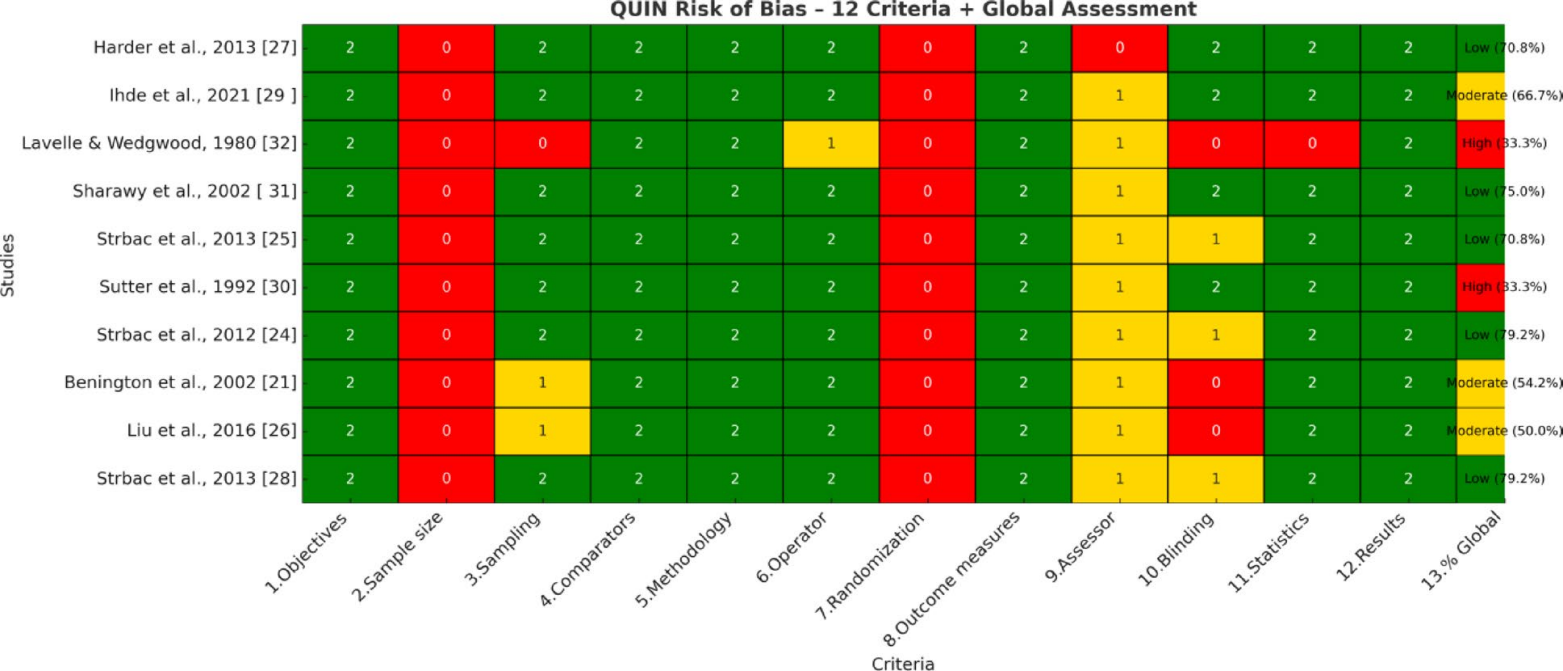
QUIN Risk of Bias Heatmap across 10 studies Green = Low (≥ 70%), Yellow = Moderate (50–69%), Red = High (< 50%)

Risk of bias was determined based on the final score, which rated the overall risk of bias for each article. The results, including all data and the risk of bias for the articles, are shown in Fig. 2.

QUIN criteria:

1. Objectives clearly stated 2. Sample size calculation 3. Sampling method 4. Comparator group(s) 5. Methodology details 6. Operator details 7. Randomization 8. Outcome measurement method 9. Outcome assessor details 10. Blinding 11. Statistical analysis 12. Results presentation.

## Results

After the first search, a total of 201 articles were found. Deduplication was able to remove 53 duplicates from the initial search (Fig. 1).

On the remaining 148 articles, a first title and abstract screening was performed to assess whether the articles were aligned with the theme and inclusion criteria, and they were retrieved for full-text analysis.

At the same time, a free terms search was performed on Google Scholar, including the terms ‘implant placement’, ‘implant bed preparation’ and ‘internal irrigation’.

42 articles were retrieved for the full-text analysis. After the full analysis, 36 articles were excluded for the final analysis. Some articles were not focusing on internal irrigation, some others were not focusing on the dentistry field (e.g., orthopedics implants bone preparation). At the end of the selection, ten in vitro articles were finally added in the qualitative analysis.

### Synthesis

The data found in the selected articles are synthesized in Table 1. All the studies performed implant bed preparation for implant placements in vitro.

**Table 1:** Summary of the results for in vitro studies

Author and year	Bone Type	Risk of bias QUIN	Measurement set-up	External irrigation		Internal irrigation		Combined irrigation	
				(ΔT)	Max temperature	(ΔT)	Max temperature	(ΔT)	Max temperature
Strabac G. et al. 2012 [24]	Bovine bone (Ribs)	Low	Custom-built thermoprobes	Maximum 2.60 °C	Below critical value	Maximum 1.48 °C	Below critical value	Maximum 1.47 °C	Below critical value
Benington C. et al. 2002 [21]	Bovine bone (mandible)	Mod	Agema Thermovision 900, infrared	Maximum, 3.1 °C	Below critical value	Maximum 3.2 °C	Below critical value	–	–
Strabac G. et al. 2013 [27]	Artificial Bovine bone specimens BoneSim	Low	Two custom-built thermoprobes	Maximum, 28.47 °C	Higher than 47 °C at 16 mm	Maximum, 25.86 °C	Higher than 47 °C at 16 mm	Maximum, 25.68 °C	Higher than 47 °C at 16 mm
Liu et al. 2016 [28]	Block fabricated DuraForm PA Plastic	Mod	Thermocouples K-type TT- Omega Engineering	46.6 °C	Near critical value (45,6 °C)	12.9 °C	Below critical value	–	–
Harder et al. 2012 [29]	Bovine bone (Ribs)	Low	Thermocouples T-type Cu-CuNi	3.9 °C	Below critical value	1.7 °C	Below critical value	–	–
Strbac et al. 2013 [30]	Artificial Bovine bone specimens BoneSim	Low	SHT-Thermoprobe	28.49 °C	Higher than 47 °C	28.30 °C	Higher than 47°	40.16 °C at	Higher than 47 °C
Ihde et al. 2021 [31]	Solid rigid polyurethane (PUR) foam block	Mod	Thermocouples and infrared (IR)	Maximum, 63.8 °C	Higher than 47 °C	Maximum, 75.4 °C	Higher than 47 °C	–	–
Sutter et al. 1992 [32]	Bovine bone (Calf humerus)	Hight	Thermal probes with plotter	35 °C	Higher than 47 °C (72 °C)	5 °C	Below critical value (42 °C)	–	–
								–	–
Sharawy et al. 2002 [33]	Porcine bone (Mandible and maxillae)	Low	Thermocouples K-type; Omega Engineering	2.8 °C	Below critical value	4.1 °C	Below critical value	–	–
Lavell e et al. 1980 [34]	Human bone (femoral)	Hight	Thermocouples connected to amplifiers	–	Higher than 47 °C (81 °C)	–	Higher than 47 °C (54 °C)	–	–

The bone type and the bone samples had different origins. The most used sample, in four studies (40%), was the bovine bone block. It was taken from ribs (2), humerus or mandible. Another material used was the artificial bone specimens, the BoneSim© in the 2 studies. Pig or human bone was used once. Synthetic materials such as polyurethane and DuraForm PA Plastic were also used once, and both are synthetic materials.

For the measurement setup, the most used method appeared to be the Thermocouples, in half of the studies (50%). These were frequently K-Type or T-Type thermocouples which operate using electric potentials to measure the temperature of the sample.

The second most used method of measurement was the thermoprobe (40%). These are contact based probes that can measure temperature.

Infrared technology was used in two studies, once alone, once combined with a thermocouple. These cameras can extrapolate temperature from the infrared radiations without requiring a contact with the surface.

The variation of temperature ΔT is shown for internal, external and combined irrigation. The greatest mean value was taken from the studies. When unavailable, it was extrapolated from graphics inside the articles.

The range of ΔT are:

- Internal irrigation from 1.48 to 75.4 °C
- External irrigation from 2.60 to 63.8 °C
- Combined irrigation from 1.47 to 40.16 °C

As for external irrigation, five studies reported maximum temperature exceeding 47 °C, while the other five reported temperatures below 47 °C.

For internal irrigation, the maximum temperature was higher than 47 °C in four studies, while in the other six studies the maximum temperature recorded was below 47 °C.

As regards combined irrigation, in two of the three studies the authors recorded values higher than 47 °C, and one study showed values below 47 °C.

In most cases (70% of studies), the peak temperature recorded for internal irrigation was lower than that for external irrigation temperature.

### Risk of bias

#### Supplementary materials

For animal studies, we could not include articles because none of them met the PICO question. They were first screened and found during the initial search. The main outcomes reported in animal studies were histologic bone healing, bone-implant contact (CBIC) and cortical bone volume (CBV), as indirect outcomes related to temperature control. We discussed in the articles found on the theme of internal irrigation in this paragraph of the review, as a ‘supplementary material’.

The risk of bias for those articles was not calculated, as they were not included in the systematic review (Table 2).

**Table 2:** Summary of the results for animal studies

Author and year	Bone Type	Outcome	External irrigation	Internal irrigation	Combined
Parr et al. 1996 [35]	Rabbit femurs	Bone healing (Weeks I-IV)	Normal healing, no signs of bone damage or healing delay (weeks I-II-III-IV)	Normal healing, no signs of bone damage or healing delay (no data for weeks 3 and 4)	–
Haider et al. 1993 [22]	Female sheep, left distal tibia	Bone-implant contact (CBIC)	Better bone implant contact on the superficial part	Better bone implant contact on the cortical deeper part	–
Trisi et al. 2013 [36]	Sheep mandible	Bone-implant contact (CBIC) and cortical bone volume (CBV)	31.024 ± 8.21% CBIC and 55.926 ± 5.15% CBV	44.02 ± 8.29% CBIC and 72.748 ± 7.44% CBV	41.198 ± 13.48% CBIC 77.384 ± 3.46% CBV

All the articles reported positive results for the internal irrigation method. The study by Parr [35] showed normal histological healing after implant bed preparation at week 1 and 2 using internal cooling method.

The study conducted by Haider was on female sheep bone, and the main outcome evaluated was the CBIC, the bone-implant contact. The role of irrigation is to reduce the bone necrosis during implant bed preparation and to give a better response from the bone tissue to implant placement. For this reason, the author focused on this parameter, which is an indirect effect of bone response after heating during implant bed preparation. The external irrigation group showed better bone-implant contact in superficial cortical bone. Internal irrigation, on the other hand, showed better results on deeper cortical bone.

Trisi et al. [36] investigated the effect of internal irrigation on sheep using the CBIC and the CBV as the main outcomes. They demonstrated the importance of irrigation in general, as the no-irrigation group showed the worst results. Combined and internal irrigation showed better results than external irrigation alone, but the difference was not statistically significant.

## Discussion

Based on our findings, internal irrigation seems to be a valid alternative to external irrigation and an effective method to reduce bone heating during implant bed preparation.

When the osteotomy exceeds 10 mm in depth, according to our findings, the internal irrigation method shows better results than external irrigation. Depending on other factors, combined irrigation may sometimes lead to a better effect on bone.

During guided surgery, when a surgical guide is used, internal irrigation seems to be more effective than external irrigation.

Implant placement is a common procedure nowadays. The procedure requires, in most cases, implant bed preparation. The most used method to perform a bone osteotomy is the use of osteotomy drills.

First studied by Eriksonn and Alberktson [6], the importance of bone heating during osteotomy is now recognized as one of the factors influencing bone healing and success in implant therapy.

Temperature threshold is important, but at the same time, the duration of the stimuli and the temperature damage are also critical factors for bone healing [36].

External or common irrigation is nowadays the main method of irrigation during the implant placement procedure [24, 37].

This can be combined with cooled or room-temperature irrigants, and even the flow rate can be changed on the setup [38].

The aim of this study was to examine the role and efficacity of internal irrigation compared to external irrigation and, when applicable, combined irrigation to reduce and control bone heating during implant bed preparation.

As concerns internal irrigation, first presented by Meyer in 1975, [23] the main point is to focus irrigation on the deeper part of the osteotomy, using internal channels to deliver irrigation to the tip of the drill and osteotomy walls, while simultaneously removing bone debris that can add some frictional resistance on the bur [21].

The disadvantages of this method include higher costs compared to traditional irrigation drills [21] and the potential for bacterial contamination inside the capillary channel of the bur. The shape of the internally cooled drill and the presence of a space inside the bur could advance the possibility of germ contamination and biotic proliferation if sterilization is inadequate. These concerns were highlighted in Proff’s et al. article, where the correct protocol of sterilization showed good results [39]. Another issue is the lower mechanical resistance due to lack of structure and density required to accommodate internal vessels, particularly in ceramic drills [40].

Combined irrigation represents the evolution of the external and internal irrigation concepts [24, 27, 29]. This method combines the effects of external and internal irrigation, providing a double surface of action on the bone, that is, external irrigation for the superficial part and the most corticalized part of the bone, and internal irrigation for the deeper part of osteotomy site [30].

When a guide for guided surgery is used, internal irrigation can help prevent the irrigation flow from being blocked by the guide itself [28]. In some cases, another irrigation channel can be added to the surgical guide [41].

In our study, we aimed to investigate the role of internal irrigation and its capacity to reduce bone temperature during osteotomy. The main method used nowadays is external irrigation. We sought to determine whether internal irrigation can control the temperature during implant placement is effectively as the external one.

The systematic review revealed no clinical studies carried out on humans. Only a few animal studies were found, but they did not match the PICO question and therefore they could not be included in the systematic’ review. For this reason, we decided to add the animal studies as Supplementary materials.

Many of the articles presented peak temperature as the main result. This is linked to the clinical explication of this parameter. The requirement in clinical practice is to avoid the critical temperature and the consequent bone necrosis.

According to the results, in 7 studies internal irrigation showed better results for the control of temperature, especially in the deeper part of osteotomy sites.

In Strabac et al.’s [24] report, internal irrigation showed better results than external irrigation, with lower temperature increment, especially in the deepest part of the osteotomy during the 16 mm depth drilling. Combined irrigation was also superior to external irrigation alone, but not to internal irrigation. All methods remained below the critical value.

In Benington et al.’s [21] study, no clinical or statistical differences were found between the two methods. Both remained below the critical threshold. The authors suggest that internal irrigation may be too expensive in clinical practice and may not result in heat reduction or to a real advantage.

Strabac’s et al.’s 2012 study [27] showed higher temperature values for external irrigation at 10 mm and 16 mm of depth. A statistical difference could be found between external and internal or combined irrigation methods. The 16 mm depth led to critical values of temperature, highlighting the effect of time and depth. Internal irrigation was more effective than combined irrigation.

In Liu et al.’s article [28], the researchers added a surgical guide to simulate the guided implant placement. The surgical guide, in the authors’ opinion, could block irrigation and reduce the cooling capacity of the traditional method. In this study, internal irrigation showed better results. External irrigation reached values near the critical limit. Adding a channel to surgical guide could help achieve adequate cooling.

Harder et al.’s study [29] investigated irrigation methods and materials. Drill material did not show any significative difference, but irrigation did. Internal irrigation showed better capacity of cooling.

In Strabac et al.’s 2013 article [28], for conical drills, internal irrigation was the most effective cooling method, even better than combined irrigation for the 10 mm depth osteotomy. At 16 mm depth, internal irrigation showed better results than external irrigation. When comparing conical drills, external irrigation provided the most effective cooling at 10—mm depth osteotomy.

This study emphasizes the withdrawal phase, where the peak temperature is reached for all the cooling methods.

Ihde et al.’s and Sharawy’s articles [31, 33] compared different drills and drilling RPM speeds. Ihde et al. reported higher temperatures variations for internally cooled drills, with values exceeding the critical threshold. The authors proposed that the larger metal volume of externally cooled drills could help absorb and better distribute heat. At the same time, in Sharawy’s article, external cooling was more effective than internal cooling. However, the study was not primarily designed to compare the two methods, and the author noted that variations in drill shape and manufacturer may have limited the validity of this comparison.”

Sutter et al.’s and Lavelle et al.’s [30, 34] studies showed better results when internal irrigation was used. Both articles were published before the 2000s. Lavelle et al. employed obsolete burs, no longer commonly used.

Sutter’s article also employed old trephine drills system from Straumann no longer used today.

The measurement setups, including thermocouples and data acquisition systems, were less accurate and powerful than current technology.

Both articles were assessed as having a high risk of bias.

When the osteotomy exceeds 10 mm in depth, our findings reveal that internal irrigation performs better than external irrigation. Sometimes, depending on other factors, combined irrigation may lead to a better effect on the bone.

Concerning measurement methods, the most commonly used was the thermocouple [28, 29, 31, 33, 34]. Although sometimes classified as a separate method, the thermoprobe is a complex instrument that can incorporate a thermocouple to measure temperature via the contact surface [24, 27, 30, 32]. Infrared thermography is a validated method [42] that appears effective for the most superficial parts of the bone.

The limitations of this review are linked to the absence of a protocol that can uniform all the variables that play a role during implant bed preparation. These include the type of material drilled and the variability between animal bone, synthetic bone, other synthetic materials, and their composition, such as spongy versus cortical bone [13]. The physical propriety of substances, like density, thermal capacity [28] and heat dissipation capacity, can lead to substantial variability in results. It has been attempted to standardize or modify materials to reduce these effects [27, 28, 30, 31]. The importance of bone density is well described in the literature. Some authors have suggested the possibility that drilling may be performed without any irrigation. This could be done in case of soft bone, like a Mish IV at low speed [10, 43].

The presence of multiple drill diameters, designs and materials for implant drills [29, 32], variations in irrigant temperatures, and drill RPM contribute to this variability [33]. At the same time, differences in measurement methods [30, 37], drilling depth [44], drilling duration [40], drill wear [14], intermittent versus continuous drilling, and the distance between the osteotomy bone wall and the probe all influence results. Thermocouples or thermoprobes are generally placed 1 mm or 2 mm from the drill. Some authors suggest that the real temperature at the bone wall during drilling may be higher than the measured value [27, 28]. Also, direct measurement on the bone wall is impossible without destroying the thermocouples or the thermoprobes [29] and in some cases only few thermocouples or measurement depths were used. In our opinion, the optimal setup uses multiple thermocouples at different depths [27, 30].

Another important limitation is the substantial heterogeneity among the included studies. Differences in bone models (animal, synthetic, or other artificial materials), drill systems and designs, drilling protocols (including speed, depth, intermittent versus continuous drilling), irrigation conditions, and temperature measurement methods significantly limit direct comparison between studies and contribute to variability in reported outcomes. In addition, all included studies were in vitro. While these models allow controlled assessment of thermal changes during implant osteotomy, they do not fully replicate clinical conditions such as blood perfusion, bone vitality, and intraoperative variability. Therefore, the clinical applicability of the findings remains limited.

For future studies, a standardized protocol reducing the risk of bias due to multiple variables should be the main goal. Further investigations will be needed to better understand the role of internal irrigation, combined with other conditions and clinical situations.

## Conclusion

According to our findings and the review of the literature, internal irrigation appears to be a potential alternative to external irrigation, especially when surgical guides are used or during deep osteotomies. In those cases, internal cooling may provide improved thermal control compared to traditional methods of irrigation.

Among the different irrigation methods, both internal and combined *irrigation* seem to demonstrate favorable cooling effects in the included studies*.*

Due to the importance of irrigation in modern implantology, it is recommended to conduct further studies with greater homogeneity among the many factors that play a role in bone heating. Additional studies on animals and humans could provide a better understanding of the role of internal irrigation in modern implantology.

## Data Availability

No new data were created or analyzed in this study. Data sharing is not applicable to this article. Acknowledgments.
